# Vision-Based Automated Recognition and 3D Localization Framework for Tower Cranes Using Far-Field Cameras

**DOI:** 10.3390/s23104851

**Published:** 2023-05-17

**Authors:** Jiyao Wang, Qilin Zhang, Bin Yang, Binghan Zhang

**Affiliations:** Department of Structural Engineering, Tongji University, 1239 Siping Road, Shanghai 200092, Chinayangbin@tongji.edu.cn (B.Y.); zhangbinghan@tongji.edu.cn (B.Z.)

**Keywords:** tower crane, computer vision, sensing system, three-dimensional localization, far-field camera

## Abstract

Tower cranes can cover most of the area of a construction site, which brings significant safety risks, including potential collisions with other entities. To address these issues, it is necessary to obtain accurate and real-time information on the orientation and location of tower cranes and hooks. As a non-invasive sensing method, computer vision-based (CVB) technology is widely applied on construction sites for object detection and three-dimensional (3D) localization. However, most existing methods mainly address the localization on the construction ground plane or rely on specific viewpoints and positions. To address these issues, this study proposes a framework for the real-time recognition and localization of tower cranes and hooks using monocular far-field cameras. The framework consists of four steps: far-field camera autocalibration using feature matching and horizon-line detection, deep learning-based segmentation of tower cranes, geometric feature reconstruction of tower cranes, and 3D localization estimation. The pose estimation of tower cranes using monocular far-field cameras with arbitrary views is the main contribution of this paper. To evaluate the proposed framework, a series of comprehensive experiments were conducted on construction sites in different scenarios and compared with ground-truth data obtained by sensors. The experimental results show that the proposed framework achieves high precision in both crane jib orientation estimation and hook position estimation, thereby contributing to the development of safety management and productivity analysis.

## 1. Introduction

A tower crane is an important component of modern construction projects. It enables the convenient and swift transportation of building resources from the supply area to the construction area, reducing construction time, lowering labor dependence, and enhancing work efficiency. With their 3 degrees of freedom (3-DOF), including the rotation of the jib, translation of the trolley, and vertical motion of the hook, tower cranes can cover the majority of the three-dimensional (3D) space on a construction site. However, due to these capabilities, tower cranes also introduce many safety hazards, including the potential risk of collision with other entities. Many studies have found that accidents involving tower cranes occur frequently on construction sites [1,2]. In 2009, 116 accidents occurred worldwide, resulting in over 44 deaths and numerous injuries [3]. In China, from 2013 to 2019, a total of 194 accidents related to tower cranes occurred, resulting in 294 deaths and 109 injuries [4]. Therefore, it is critical to obtain accurate information on localization to build a real-time location system (RTLS) for tower cranes.

Previously, traditional tower crane monitoring methods relied on manual judgment or contact sensors that transmitted information through a wireless sensor network (WSN). Contact sensors are a type of invasive sensing method that is susceptible to noise interference [5]. In recent years, with the development of information technology, the automation of 3D localization has become possible on construction sites. Information technologies, such as global positioning systems (GPS) [6], radio frequency identification (RFID) [7], ultra-wideband (UWB) [8], and 3D point clouds based on laser scanning [9], have been widely used on construction sites for entity localization. However, they also have limitations. For example, high-precision GPS and laser scanning methods [10] require a huge upfront investment in additional equipment. UWB and passive RFID are mainly used for short sensing distances [11]. Sensing devices are fragile and require frequent maintenance, which increases costs and time. Generally speaking, these sensing methods are not suitable for wide outdoor scenes.

As a non-invasive sensing method, vision can minimize interference with construction. Cameras have advantages such as a wide field of view, simple maintenance, and low cost. Computer vision-based (CVB) technology makes image analysis simple and it is widely used for object detection on construction sites [12]. With the development of deep learning (DL), a convolutional neural network (CNN) was proposed in 1998 [13]. In 2012, AlexNet was applied to CNN for image classification, achieving significant results in the ImageNet competition [14]. CNN-based object detection algorithms have achieved excellent detection results. CVB technologies are widely used on construction sites for the 3D localization of mobile resources such as workers [15], trucks [16,17], and components [18]. However, for monocular cameras, image detection technology can only provide the coordinates of objects in the two-dimensional (2D) pixel plane, resulting in a loss of the depth relationship in 3D space. Monocular vision-based depth estimation often requires additional prior knowledge, such as information about the extrinsic parameters, texture, and lighting [19]. Most of the existing monocular vision-based 3D localization methods focus on ground planes by using affine transformation [16], estimating 3D spatial proximity from 2D pixel coordinates. However, for aerial objects, there are currently limited solutions available for position estimation. For the 3D localization of crane hooks, top-view cameras mounted on jibs are usually utilized [20,21]. However, this introduces additional installation and interference issues, as well as a narrow field of view.

Therefore, this research aims to address the problem of the 3D localization of tower cranes and hooks using monocular far-field surveillance cameras. The main contribution of this paper is to achieve precise tower crane pose estimation and hook 3D localization from a distant perspective using a monocular surveillance camera. This paper proposes a framework consisting of four steps. The first step is the autocalibration of the camera’s extrinsic parameters using feature matching and horizon-line detection algorithms. The second step is the use of the Mask-RCNN instance segmentation algorithm to identify the tower crane and hook. The third step employs a hybrid method, combining a contour extraction algorithm with a line segment detection (LSD) algorithm, for reconstructing the geometric features of the tower crane. The fourth step involves estimating the jib orientation and hook localization using a backpropagation neural network (BPNN) based on the geometric features. The rest of the paper is organized as follows. Section 2 presents a literature review of studies of vision-based methods on construction sites. Section 3 illustrates the framework of the proposed method. Section 4 presents the results of a case study. Section 5 presents the discussion and conclusions.

## 2. Related Works

### 2.1. Vision-Based Object Detection on Construction Sites

Object detection and tracking are important prerequisites for the analysis of construction activities. By extracting information about construction entities, such as workers, materials, and machinery, further work can be carried out. With the development of computer technology, vision-based object detection and tracking methods are widely used on construction sites. Early research mainly focused on utilizing pixel features such as color; hue, saturation, value (HSV); shape context; and histogram of orientation gradients (HOG). The HOG with Harri-like features algorithm has been used to detect trucks on construction sites from continuous video streams [22].

With the rapid development of DL, in 2014, a region-based convolutional neural network (RCNN) was proposed for object detection [23]. The method is to extract region proposals, compute feature vectors using a CNN, and, finally, classify every region. Subsequently, Fast-RCNN [24], Faster-RCNN [25], and Mask-RCNN [26] algorithms were proposed. Since then, CNN-based object detection methods have been applied to various fields due to their accuracy and convenience. Ref. [27] used Mask-RCNN to identify the relationship between workers and concrete supports as a way to determine unsafe behavior. Ref. [28] proposed a three-CNN framework to accurately identify excavator activity. In another study, Mask-RCNN was combined with the multi-object tracking algorithm DeepSORT to identify wall installation progress on construction sites [29].

In order to further analyze object pose and orientation, instance segmentation is necessary because it can facilitate the reconstruction of geometric features. Compared with other algorithms, Mask-RCNN achieves pixel-level instance segmentation, which is of great significance for analyzing object pose in a more refined approach. In this paper, Mask-RCNN is chosen as the instance segmentation algorithm for tower cranes and hooks.

### 2.2. 3D Localization on Construction Sites

There are two types of vision-based 3D localization. The first is stereo vision and the second is monocular vision. There are some limitations of stereo vision. Stereo vision with a fixed baseline has a short visual range and cannot be applied to far-field vision. Stereo vision with a long baseline relies on two or more cameras, which are positioned far apart, that require frequent manual fine-tuning to maintain the extrinsic parameters [30]. Monocular vision-based 3D localization has also been explored in the field of computer vision and is referred to as monocular depth estimation or monocular 3D reconstruction. To achieve this, additional information is provided in advance, such as shape-from-template (SfT) [31] and illumination details [32]. The above methods, which are often used for indoor scenes, are not suitable for construction sites due to their limited range of estimation.

On construction sites, a common approach for estimating the proximity of surrounding entities is to mount cameras on mobile resources such as vehicles and workers using first-person view (FPV). For example, the global localization of mobile robots was achieved using monocular cameras by matching arbitrarily shaped (AS) features on the ceiling [33] and by segmenting environmental elements based on the real-time semantics of ENet [34] combined with perspective transformation [35]. A similar study focused on worker localization and proposed a worker global localization approach using an FPV camera [36]. In [37], the existing monocular camera in a heavy vehicle was utilized to calculate the relative positions of workers. FPV images can be used for the distance estimation of nearby objects but the field of view is narrow and it is difficult to perceive larger scenes. Unmanned aerial vehicles (UAVs) can also be used for localization due to their wide range of views. A framework for proximity estimation between workers and machines was presented, which involved capturing images of the field from UAVs in an approximately vertical manner [38]. On this basis, a social generative adversarial network (GAN) was utilized to develop active trajectory prediction [39]. In addition, 3D localization can be achieved using cameras installed in high positions, which are generally referred to as far-field surveillance cameras, the most common type of camera on construction sites. The term far-field cameras was used in [40,41,42] to describe these cameras. Ref. [43] transformed the workers’ bounding box and the ground into vertical projections following a CNN-based detection method. There are also some studies that utilized deep learning for proximity estimation on construction planes [44]. For mechanical equipment and building materials, 3D localization can be carried out using methods based on the rigid structure [45].

In general, the challenge of localization based on monocular vision lies in obtaining the extrinsic parameters of the camera. Therefore, most of the studies have focused on localization on construction ground planes [42,43,44,45,46], where it is convenient to perform perspective transformation and estimate the proximity of objects in three-dimensional space through two-dimensional pixel coordinates. In contrast, there is relatively less research on the 3D localization of objects in higher positions.

### 2.3. Vision-Based 3D Localization for Tower Cranes

A tower crane is specialized machinery with a long jib, which has 3-DOF, including the jib rotation, trolley movement, and hook vertical movement. Therefore, for the 3D localization of a tower crane, it cannot be treated as a single point, neglecting its 3D attitude. A common approach is to mount a camera on the jib or trolley and capture images of the hook or the hoisting load from above. Feature-based recognition algorithms [47,48,49,50,51] or deep learning-based detection algorithms [52] are usually used to identify the hooks. Some studies have conducted 3D reconstruction of the working ground of tower cranes. In [53], by installing a camera at the end of the jib, the characteristics of different SLAM algorithms for reconstruction were compared. Ref. [54] proposed a hybrid framework combining monocular vision with point clouds for the 3D reconstruction of tower crane working sites.

The advantages of these methods are that the camera has an approximately perpendicular viewpoint to the ground and is relatively close to the hook or hoisting load, which enables the estimation of the 3D coordinates using 2D pixels, simplifies the calculation method, and makes it easier to obtain the extrinsic parameters of the camera. There are also limitations, such as the inability to perceive the overall environment around the crane and the narrow field of view, which introduce additional issues due to the installation of extra equipment.

Another study identified the jib rotation angle using a vision-based method [55]. The approach used a threshold segmentation algorithm to extract crane jib pixels and then matched them with the silhouette of a 3D model using Kalman filtering in order to estimate the rotation angle of the crane jib. The recognition of the excavator posture also provided inspiration for this research. Ref. [56] used a vision-based algorithm to accurately extract the skeletons of the various parts of the excavator such as the body, dipper, boom, and bucket. However, further analysis of 3D pose estimation was not conducted. Ref. [57] estimated the orientation and position of excavators using monocular cameras by mounting markers on the excavators. To better understand monocular vision-based 3D localization, Table 1 summarizes the limitations of the localization methods used for aerial objects and ground objects.

To conclude, the difficulty of monocular vision-based 3D localization lies in acquiring the extrinsic parameters. Most 3D localization research considers the entity as a point, ignoring the size and pose. For accurate pose estimation of large machinery, a more detailed analysis is necessary. Based on these limitations, it is critical to further explore the 3D localization of monocular vision.

## 3. Framework and Design

The objective of this study is to propose a framework for the automatic 3D localization of crane jibs and hooks using monocular far-field surveillance cameras. To realize this goal, three core problems need to be addressed:

- how to accurately acquire the extrinsic parameters such as the camera height and Euler angles, given that far-field cameras are installed in high positions;
- how to extract the geometric features of a tower crane and its hook from segmented images;
- how to infer the crane’s rotation angle and hook position from the geometric features.

In this framework, the instance segmentation of the tower crane and hook is the basis for 3D localization. Since the Mask-RCNN algorithm implements instance segmentation, which is widely used in construction scenarios, it is used in this paper.

The framework of this paper is divided into four steps as follows:

1. A feature-based matching algorithm and a horizon line detection algorithm are used for the computation of the camera’s extrinsic parameters. The feature-matching algorithm is used to calculate the translation vector. The horizon-line detection algorithm is utilized to calculate the Euler rotation angle. This step is used for initialization and is only loaded once throughout the entire process.
2. A pre-trained Mask-RCNN model is used for the instance segmentation of the crane jib and hook.
3. Based on the pixels segmented in step 2, a hybrid algorithm combining contour extraction with a mobile line-segment detector (MLSD) is used to reconstruct the geometric features.
4. Once the camera’s extrinsic parameters are obtained in step 1, a BPNN is trained for the estimation of the jib rotation angle. Based on the geometric features obtained in step 3, the rotation angle of the jib and the hook position are predicted.

**Figure 1:**
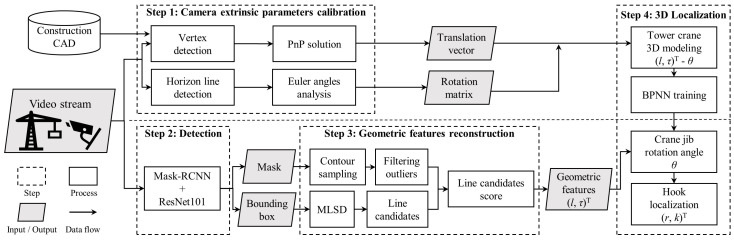
Overview of the 3D localization framework.

Figure 1 illustrates the structure and process of the entire framework. The steps are described in detail in the following subsections.

### 3.1. Calibration of Camera’s Extrinsic Parameters 

For 3D localization using monocular vision, the biggest challenge is obtaining the extrinsic parameters of the camera, especially for far-field surveillance cameras installed in high positions, which makes it hard to measure various parameters. This subsection addresses how to obtain the extrinsic parameters of the far-field camera.

**Figure 2:**
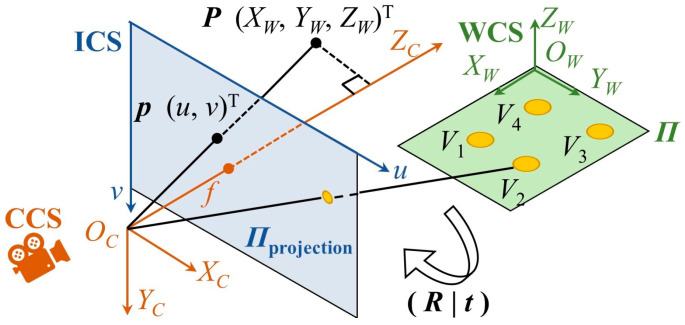
Camera pinhole imaging model.

The left side of Figure 2 shows a pinhole imaging model of a camera [58]. There are three coordinate systems: the world coordinate system (WCS), the camera coordinate system (CCS), and the imaging coordinate system (ICS). The origin of the CCS is at the optical center, where the $Z_{C}$-axis is along the camera’s optical direction. The position of any point P has the coordinates ${\left(\begin{array}{ccc} X_{W} & Y_{W} & Z_{W} \end{array}\right)}^{T}$ under the WCS, ${\left(\begin{array}{ccc} X_{C} & Y_{C} & Z_{C} \end{array}\right)}^{T}$ CCS, and ${\left(\begin{array}{cc} u & v \end{array}\right)}^{T}$ ICS, and the transformation equations are as follows:

(1) $$Z_{C}\left(\begin{array}{c} u\\ v\\ 1 \end{array}\right)=\left(\begin{array}{ccc} f_{x} & 0 & u_{0}\\ 0 & f_{y} & v_{0}\\ 0 & 0 & 1 \end{array}\right)\left(\begin{array}{cc} R & t \end{array}\right)\left(\begin{array}{c} X_{W}\\ Y_{W}\\ Z_{W}\\ 1 \end{array}\right),$$

(2) $$R=R_{Z}R_{Y}R_{X}=\left(\begin{array}{ccc} r_{11} & r_{12} & r_{13}\\ r_{21} & r_{22} & r_{23}\\ r_{31} & r_{32} & r_{33} \end{array}\right),$$

(3) $$R_{X}=\left(\begin{array}{ccc} 1 & 0 & 0\\ 0 & \cos\alpha & -\sin\alpha \\ 0 & \sin\alpha & \cos\alpha \end{array}\right),$$

(4) $$R_{Y}=\left(\begin{array}{ccc} \cos\beta & 0 & -\sin\beta \\ 0 & 1 & 0\\ \sin\beta & 0 & \cos\beta \end{array}\right),$$

(5) $$R_{Z}=\left(\begin{array}{ccc} \cos\gamma & -\sin\gamma & 0\\ \sin\gamma & \cos\gamma & 0\\ 0 & 0 & 1 \end{array}\right),$$

where R is the rotation matrix from the WCS to CCS; t is the translation vector from the WCS to CCS; $f_{x}$, $f_{y}$ are the pixel scale factors in the u-direction and v-direction, respectively; ${\left(\begin{array}{cc} u_{0} & v_{0} \end{array}\right)}^{T}$ are the pixel plane center coordinates; and $R_{X}$, $R_{Y}$, and $R_{Z}$ are the rotation matrices on the $X_{W}$-axis, $Y_{W}$-axis, and $Z_{W}$-axis, respectively. The Euler rotation angles are, respectively, α, β, and γ.

#### 3.1.1. Translation Vector

In theory, when a camera is installed on a construction site, its coordinates on the construction plane can be directly obtained from the CAD drawings. However, in reality, there are differences between the actual construction site and the CAD drawings. Therefore, directly obtaining the coordinates from the CAD drawings can cause errors and distortion in the 3D estimation. Therefore, the calculation of the translation vector t using homography was considered. The Perspective-n-Point (PnP) problem refers to estimating the pose of a camera given a set of n 3D points in the real world and the corresponding 2D projections on the image [59]. The right side of Figure 2 shows a PnP problem. On construction sites, there are many landmarks of prior knowledge that can be used for PnP estimation.

**Figure 3:**
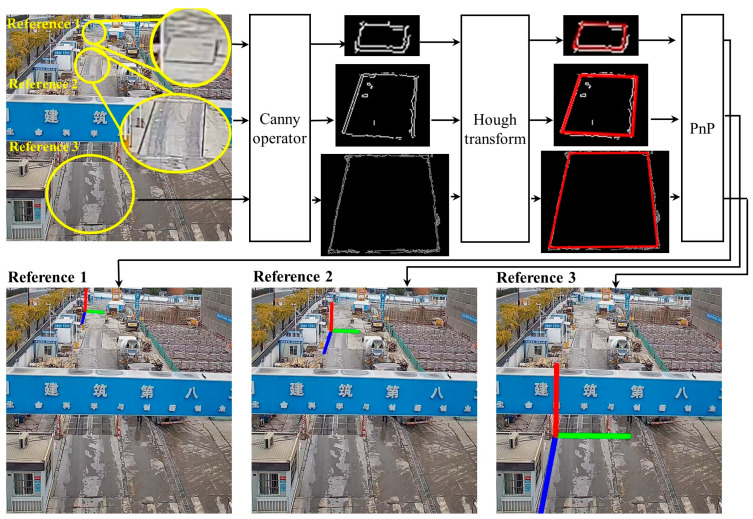
Camera translation vector calculation.

A detailed illustration of this step is shown in Figure 3. First, the corresponding feature objects are registered in topological logical order from the CAD drawings in a hash map. A hash map is a data structure that allows efficient queries in *O*(1) time complexity. Next, the Canny algorithm is used to extract the edge points [60], not the line segments. The Gaussian filter and Sobel operator are used to perform edge detection. Then, line detection is conducted using the Hough transform [61,62]. Finally, translation vector t can be obtained based on the PnP pose estimation.

#### 3.1.2. Euler Rotation Angles

**Figure 4:**
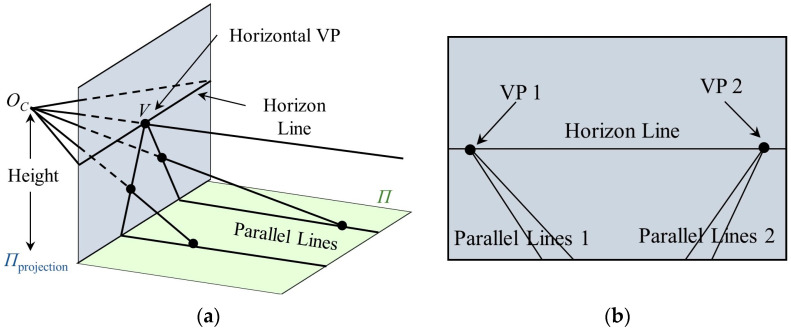
Horizon-line model of camera. (**a**) Horizon line in 3D; (**b**) Horizon line in 2D.

The angle of rotation around the $Z_{C}$-axis is called the roll angle γ; the angle of rotation around the $Y_{C}$-axis is called the yaw angle β; and the angle of rotation around the $X_{C}$-axis is called the pitch angle α. As shown in Figure 4a, there is a set of parallel lines on the ground plane, whose vanishing point (VP) on the projection plane is V. In natural environments, there are often multiple sets of parallel lines, and the line composed of the VPs is called the horizon line [63], as shown in Figure 4b.

There have been many studies aimed at extracting the horizon line from images. In [64], a CNN-based method was implemented to obtain the probability distribution of horizon-line candidates. Subsequently, in [65], an accurate method for detecting the horizon line was proposed. The aim was to generate a continuous probability distribution of the horizon line through GoogLeNet, select the horizontal VPs on the horizon-line candidates, and finally select the best horizon line based on the score of horizontal VPs. Therefore, in this paper, the methods of [64,65] are adopted to accurately detect the horizon line.

**Figure 5:**
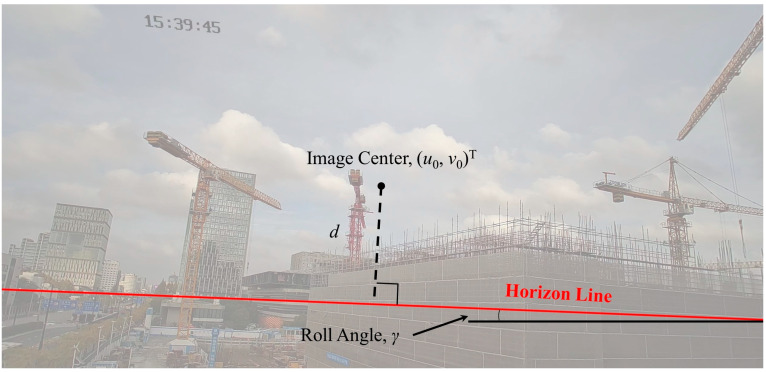
Horizon-line detection.

**Figure 6:**
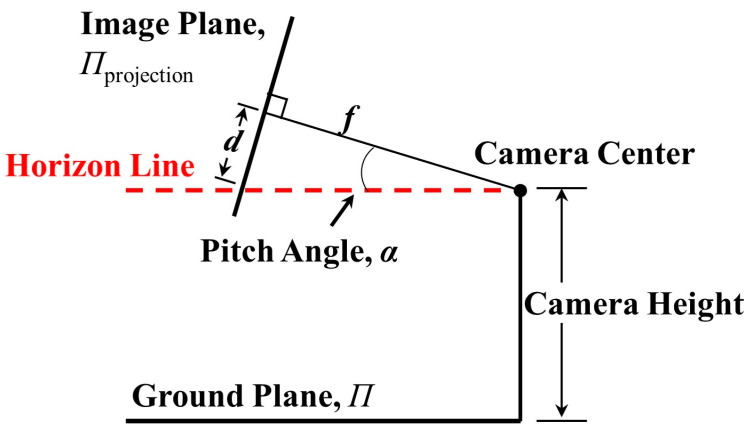
Side view of horizon-line detection model.

Figure 5 shows the horizon-line detection and gives the roll angle γ. Figure 6 shows the side view of the horizon detection pattern and gives the pitch angle α. The yaw angle β is defined as the angle between the optical axis and the tower crane. The calculation equations are as follows:

(6) $$\alpha =\arctan\frac{d}{f},$$

(7) $$\beta =\arctan\frac{o_{\mathrm{tower}}}{f},$$

(8) $$\gamma =\arctan\frac{y_{2}-y_{1}}{x_{2}-x_{1}},$$

where d is the vertical distance between the image center and horizon line; f is the focal length of the camera; $o_{\mathrm{tower}}$ is the offset between the image center and tower crane; and ${\left(\begin{array}{cc} x_{1} & y_{1} \end{array}\right)}^{T}$, ${\left(\begin{array}{cc} x_{2} & y_{2} \end{array}\right)}^{T}$ are the left and right points of the horizon line, respectively.

### 3.2. Detection and Segmentation Based on Mask-RCNN

Object detection and instance segmentation are the foundations of 3D localization. The most common use of object detection algorithms is to generate a bounding box. This approach is reasonable for small entities such as workers [66] and prefabricated components [67]. However, tower cranes have characteristics such as a long jib length so estimating the pose by only generating bounding boxes is insufficient. In order to accurately analyze the posture of a tower crane, it is necessary to segment the pixels of the tower crane.

**Figure 7:**
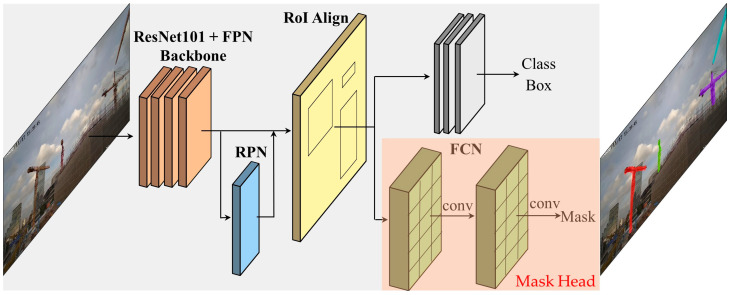
The architecture of Mask-RCNN.

The Mask-RCNN algorithm can effectively detect targets and output high-quality pixel masks. The architecture of Mask-RCNN is shown in Figure 7. A ResNet-101 network and feature pyramid network (FPN) are used as the backbone. A region proposal network (RPN) is used to obtain the region of interest (RoI). Mask-RCNN uses RoI Align to accurately calculate the pixel information. The last step is divided into two stages. The first generates the category and bounding box. The second uses a fully convolutional network (FCN) to generate the masks.

### 3.3. Geometric Feature Reconstruction

After obtaining the pixel masks, the most important task is to reconstruct the geometric features of the tower crane, which is a morphological problem. In theory, it is reasonable to consider all of the masks as the BPNN input. However, two problems arise when regarding the pixel mask as a feature map:

1. the mask is a large matrix, which leads to significant processing time and makes it difficult to achieve real-time performance;
2. the mask does not completely cover the entity for various reasons, leading to an increase in estimation errors.

**Figure 8:**
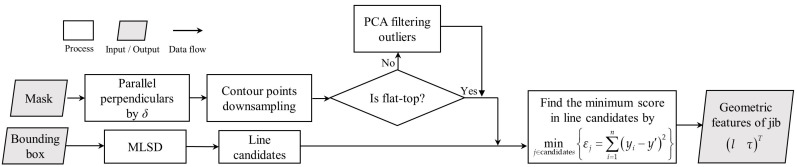
The detailed procedure for reconstructing the geometric features.

In this study, the tower crane can be abstracted as a rigid model. Rigidity means that knowing the position of any one point can provide information about its motion state, i.e., the relative position between each point will not change. Therefore, the projected line segment of the jib on the image is selected as the geometric feature ${\left(\begin{array}{cc} l & \tau \end{array}\right)}^{T}$, where l is the length of the projected line and τ is the angle of the projected line segment to the u-axis. A mobile line segment detector (MLSD) [68] is applied to extract straight lines in this paper. The specific process is shown in Figure 8, which is divided into two steps: contour extraction and line segment detection based on MLSD.

#### 3.3.1. Extraction of Contour Points

To address the first issue mentioned above, we propose a downsampling method to reduce the number of points on the mask contour. First, a series of perpendicular lines with a fixed distance δ, usually between 0.01 and 0.02 of the width of the bounding box, are given within the bounding box. Then, the tower crane pixel mask is intersected with these perpendicular lines to obtain a series of intersection points.

**Figure 9:**
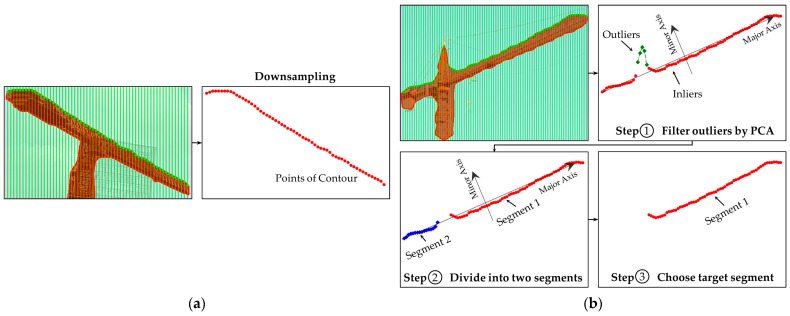
Contour point extraction process for two different types of tower cranes: (**a**) flat-top tower crane; (**b**) non-flat-top tower crane.

Figure 9 shows the contour point extraction process for two types of tower cranes. If the tower crane is not a flat-top tower crane, as shown in Figure 9b, outliers are also detected, which we do not need. Therefore, we filter out the outliers using Principal Component Analysis (PCA). The number of points extracted from the contour is denoted as m.

#### 3.3.2. Line Segment Detection and Geometric Feature Reconstruction Using MLSD

An MLSD is a lightweight and fast deep learning-based line detection method. It proposes an extremely effective architecture that minimizes the backbone network and eliminates the typical multi-module line segment prediction process. The segments of line segment (SoL) augmentation subdivides a line into multiple subcomponents. The geometric relationship loss allows the model to obtain additional geometric clues from the matching loss.

**Figure 10:**
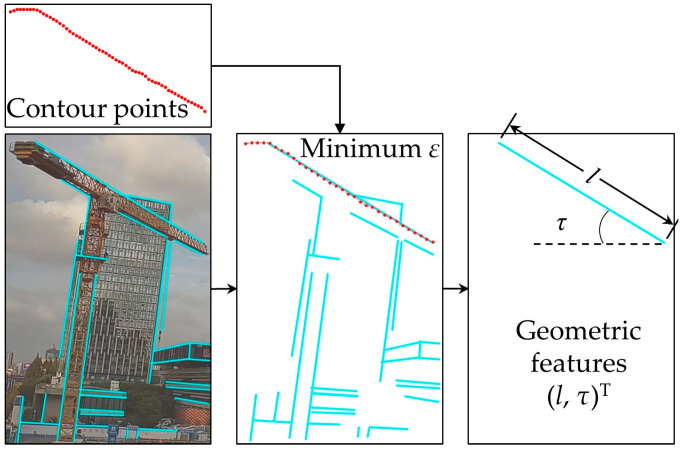
Geometric feature reconstruction using the MLSD.

We only analyze the image inside the bounding box to speed up the computation. The overall process is shown in Figure 10. First, we use a pre-trained MLSD model to detect a series of line candidates, where the number is denoted as n. Scores and thresholds are set when using the MLSD to preliminarily filter out shorter line segments. Then, we calculate the sum of the squared residuals of all the line candidates and the mask contour points:

(9) $$\underset{j\in \mathrm{candidate}s}{\min}\left\{\varepsilon_{j}=\sum_{i=1}^{m}{\left(y_{i}-y^{\prime }\right)}^{2}\right\},$$

where j represents the line candidates, $\varepsilon_{j}$ is the sum of the squared residuals, $y_{i}$ is the y coordinate of the i-th contour point, and $y^{\prime }$ is the y coordinate of the i-th point corresponding to the j-th line. The line candidate with the minimum sum of squared residuals is identified as the true contour line of the tower crane. The geometric feature ${\left(\begin{array}{cc} l & \tau \end{array}\right)}^{T}$ is also provided. ${\left(\begin{array}{cc} l & \tau \end{array}\right)}^{T}$ is chosen as the line feature because it comprehensively considers all the pixel information of the edge contour, which can maximize the elimination of outliers and interference from the horizontal and vertical directions, resulting in more accurate prediction results.

As shown in Figure 10, we can see that a significant number of line candidates have no relation to the tower crane. The number of points extracted from the contour is m so the time complexity is $O\left(m\times n\right)$. In order to speed this up, we utilize the PCA results to quickly filter out the unrelated line candidates. The center point of PCA is ${\left(\begin{array}{cc} x_{\mathrm{pca}} & y_{\mathrm{pca}} \end{array}\right)}^{T}$ and the angle of the major principal is ω. By using PCA, we can set a confidence ellipse. Line candidates whose absolute difference with the major principal is greater than ξ and whose center point is outside the confidence ellipse can be filtered out, as shown in Equation (10):

(10) $$\left\{\begin{array}{c} {\left(\begin{array}{cc} x_{cj} & y_{cj} \end{array}\right)}^{T}\notin \left\{\begin{array}{cc} \mathrm{confidence} & \mathrm{ellipse} \end{array}\right\}.\\ \left|\omega -\omega_{j}\right|>\xi \end{array}\right.\;,$$

where ${\left(\begin{array}{cc} x_{cj} & y_{cj} \end{array}\right)}^{T}$ is the center of the j-th line candidate and $\omega_{j}$ is the angle of the j-th line candidate. Therefore, this enables fast filtering, leaving only a few remaining line candidates (usually less than five). The time complexity is reduced from $O\left(m\times n\right)$ to $O\left(m+n\right)$.

### 3.4. Estimation of 3D Localization 

The 3D localization of the tower crane consists of two steps: the estimation of the jib rotation angle and the estimation of the hook position.

#### 3.4.1. Estimation of Jib Rotation Angle

**Figure 11:**
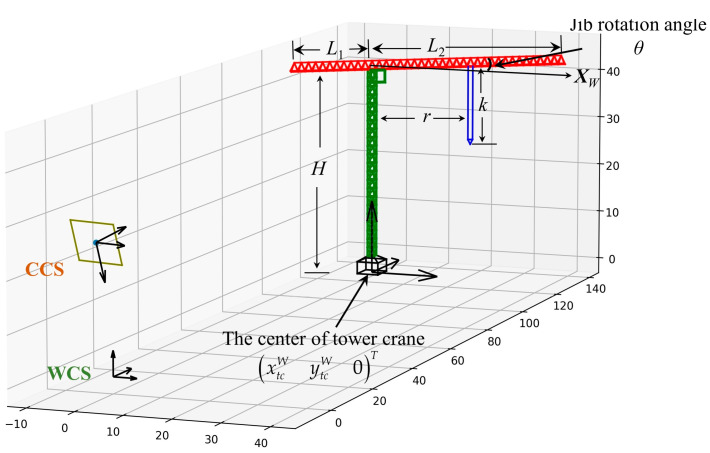
The 3D model of the tower crane.

In the first step, we obtain the camera’s height, roll angle γ, yaw angle β, and pitch angle α, and the extrinsic parameter matrices of the camera can thus be established. In addition, the parameters of the target tower crane such as the body height and jib length can be obtained from the factory information, which is a priori semantic knowledge. Therefore, the relative positions of the camera and tower crane can be determined, as shown in Figure 11.

The tower crane is abstracted as a 3D rigid model, with center coordinates of ${\left(\begin{array}{ccc} x_{tc}^{W} & y_{tc}^{W} & 0 \end{array}\right)}^{T}$, a body height of H, a jib length of $L_{1}$ and $L_{2}$, and a jib rotation angle θ relative to the $X_{W}$-axis of the WSC. According to the camera model, there is only one variable parameter, θ. In addition, based on the projective geometry principle, the projection of a straight line remains a straight line. Therefore, the geometric features depend only on θ:

(11) $${\left(\begin{array}{cc} l & \tau \end{array}\right)}^{T}\propto \theta ,$$

There exists a nonlinear relationship between the geometric features ${\left(\begin{array}{cc} l & \tau \end{array}\right)}^{T}$ and the jib rotation angle θ, We use a backpropagation neural network (BPNN) to estimate this relationship. A BPNN is a multi-layer feedforward network trained using the error backpropagation algorithm, which can learn and store a large number of input–output pattern mappings, without the need to reveal the mathematical equations. The backpropagation algorithm is used to compute the gradient of the loss function with respect to each weight for a single input–output instance using the chain rule to calculate the gradient of one layer at a time and iteratively propagating backwards from the last layer. This approach avoids redundant calculations of intermediate terms in the chain rule.

**Figure 12:**
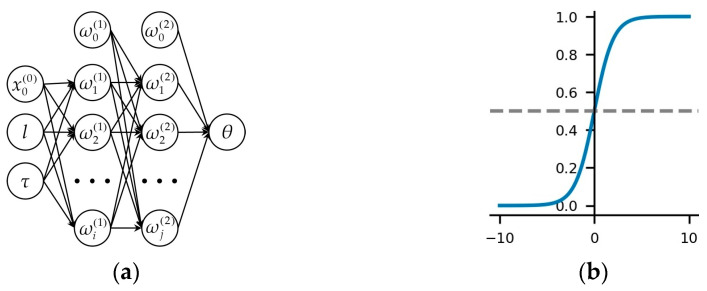
The BPNN in this study: (**a**) The architecture of the BPNN; (**b**) Sigmoid function.

In this study, we adopted a four-layer BPNN, as shown in Figure 12a, with the input layer consisting of ${\left(\begin{array}{cc} l & \tau \end{array}\right)}^{T}$ and the output layer consisting of the jib rotation angle θ. The hidden layer contains two layers with 10 and 5 neurons, respectively. The sigmoid function is chosen as the activation function, which exhibits excellent nonlinearity.

Then, a dataset consisting of ${\left(\begin{array}{cc} l & \tau \end{array}\right)}^{T}$ and the jib rotation angle θ is generated using Equations (1)–(5) and trained using the BPNN. The ratio of the training set and validation set is 7:3. Finally, the actual jib angle θ is estimated using the geometric features obtained in step 3.

#### 3.4.2. Estimation of Hook Localization

In the tower crane 3D model shown in Figure 11, the distance between the trolley and the tower body is denoted as r and the length of the hoisting rope is denoted as k. The hook bounding box is obtained using Mask-RCNN and the center point of the bounding box is used to replace the hook. The jib rotation angle θ was obtained in the previous subsection. The imaging point of the hook on the image is ${\left(\begin{array}{cc} u_{h} & v_{h} \end{array}\right)}^{T}$ and the coordinates of the hook in the WSC are ${\left(\begin{array}{ccc} x_{h}^{W} & y_{h}^{W} & z_{h}^{W} \end{array}\right)}^{T}$. Therefore, according to Equations (1)–(5), r and k can be calculated as follows:

(12) $$\begin{array}{l} \left(\begin{array}{cc} r_{11}\cos\left(\theta \right)+r_{12}\sin\left(\theta \right)-\frac{u_{h}-u_{0}}{f_{x}}\left(r_{31}\cos\left(\theta \right)+r_{32}\sin\left(\theta \right)\right) & \frac{u_{h}-u_{0}}{f_{x}}r_{33}-r_{13}\\ r_{21}\cos\left(\theta \right)+r_{22}\sin\left(\theta \right)-\frac{v_{h}-v_{0}}{f_{y}}\left(r_{31}\cos\left(\theta \right)+r_{32}\sin\left(\theta \right)\right) & \frac{v_{h}-v_{0}}{f_{y}}r_{33}-r_{23} \end{array}\right)\left(\begin{array}{c} r\\ k \end{array}\right)=\\ \left(\begin{array}{c} \frac{u_{h}-u_{0}}{f_{x}}\\ \frac{v_{h}-v_{0}}{f_{y}} \end{array}\right)\left(\begin{array}{cccc} r_{31} & r_{32} & r_{33} & t_{3} \end{array}\right)\left(\begin{array}{c} x_{tc}^{W}\\ y_{tc}^{W}\\ H\\ 1 \end{array}\right)-\left(\begin{array}{cccc} r_{11} & r_{12} & r_{13} & t_{1}\\ r_{21} & r_{22} & r_{23} & t_{2} \end{array}\right)\left(\begin{array}{c} x_{tc}^{W}\\ y_{tc}^{W}\\ H\\ 1 \end{array}\right) \end{array},$$

## 4. Implementation and Results

The proposed framework was tested on a real large-scale construction site by installing a fixed-position camera at the edge of the site to provide far-field monitoring of one of the tower cranes. The camera was a Hikvision camera with a resolution of 2560 × 1440 and H.264 video encoding, which was installed at a height of approximately 11 m. The system ran on a computer equipped with an Intel Core i9-10900X CPU, operating at 3.70 GHz, with 64 GB of memory and four NVIDIA GeForce RTX 2080Ti graphics processing units (GPUs). This framework utilized advanced computer vision algorithms, such as Mask-RCNN (https://github.com/matterport/Mask_RCNN), gc-horizon-detector (https://github.com/viibridges/gc-horizon-detector), and MLSD (https://github.com/navervision/mlsd), which were forked from the master branches of open source code repositories on GitHub and accessed on 7 October 2022. Other algorithms, such as Canny, Hough, and PnP, were integrated into OpenCV version 4.4.0.

The following three subsections demonstrate the performance of the algorithms used in each module, including the accuracy of camera self-calibration, an evaluation of the accuracy and speed of Mask-RCNN-based detection, and a performance evaluation of the geometric feature reconstruction and 3D localization estimation.

### 4.1. Accuracy of Camera Self-Calibration

The surveillance camera was installed at a fixed location on the boundary of the construction site. During the initialization stage shown in step 1, the camera was slowly rotated in various directions to search for pre-defined calibration references on the construction site. The camera height, h, is more important than its x and y coordinates. We defined the camera’s vertical downward direction as the origin of the WCS and the vertical upward direction as the $Z_{W}$-axis. Table 2 lists the results of the camera’s extrinsic parameters. The results indicate that the estimated camera height had a percentage error (PE) of 1.90% compared to the ground-truth value, which is quite close for a large construction site. The PEs for the camera’s yaw angle, pitch angle, and roll angle were 4.14%, 3.79%, and 1.48%, respectively, with the error in the yaw angle being the largest. However, according to projective geometry, the roll angle has a greater impact on the measurement accuracy compared to the yaw angle.

### 4.2. Performance of Detection and Segmentation

**Figure 13:**
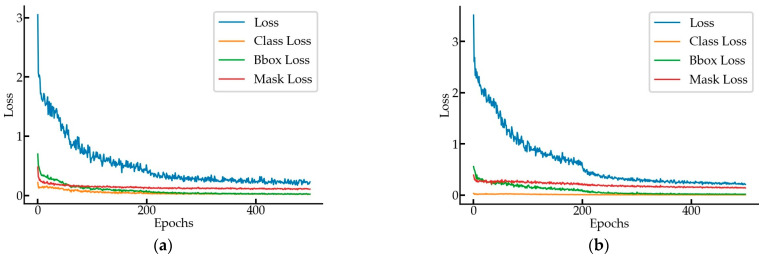
The loss curves for training the (**a**) tower crane; (**b**) hook.

A Mask-RCNN and ResNet101 backbone was utilized to perform the detection and segmentation of tower cranes and hooks from videos. The training set was composed based on the MOCS dataset provided in [69] and the images taken on the construction site. The tower crane training set contained 4000 images and the validation set contained 500 images, with a ratio of 8:1. The hook training set contained 2200 images and the validation set contained 250 images, with a ratio of 8.8:1. Mask-RCNN was trained with a learning rate of 0.001, learning momentum of 0.90, and weight decay of 0.0001. The training process used a joint training strategy for a total of 500 epochs, including 40 epochs for the RPN, classifier, and mask heads of the network; 160 epochs for ResNet stage 4 and up; and 300 epochs for all of the layers, with 100 steps per epoch. The speed for inference was 6.23 frames per second (FPS) in GPU mode. From the training loss–epoch curve in Figure 13, we can see that the total loss value dropped fast in the first 200 epochs, decreased slowly in the 200th to 400th epochs, and tended to level off after the 400th epoch.

**Figure 14:**
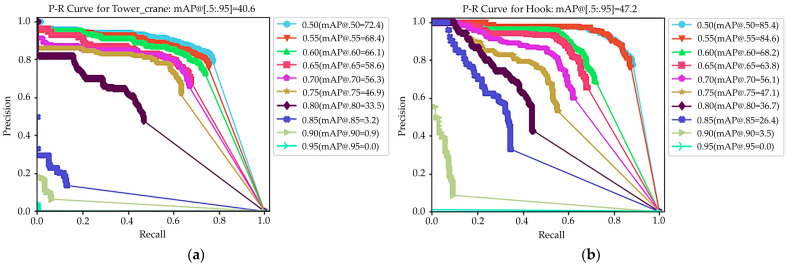
P–R curves for Mask-RCNN: (**a**) tower crane; (**b**) hook.

To evaluate the performance of Mask-RCNN, we used the metrics of the MS-COCO dataset [70]. One metric was the intersection over union (IoU) between the predicted bounding box and the ground-truth bounding box. The calculations for the precision and recall are as follows:

(13) $$\mathrm{Precision}=\frac{\mathrm{TP}}{\mathrm{TP}+\mathrm{FP}},$$

(14) $$\mathrm{Recall}=\frac{\mathrm{TP}}{\mathrm{TP}+\mathrm{FN}},$$

where TP is true positive, FP is false positive, and FN is false negative. The mean average precision (mAP) is also an evaluation metric used in MS-COCO. Figure 14 illustrates the precision–recall curves for the tower crane and hook detection at 10 IoU levels ranging from 0.50 to 0.95.

Table 3 presents the mAP values of the tower crane and hook at 0.50 and 0.75 IoU levels, as well as the benchmark results from Ref. [69]. It can be seen that the training results in this study were close to the benchmark results, indicating the good performance of the Mask-RCNN trained in this study. For the hook, both the AP with a 0.50 IoU and mAP metrics were higher than those of the benchmark.

**Figure 15:**
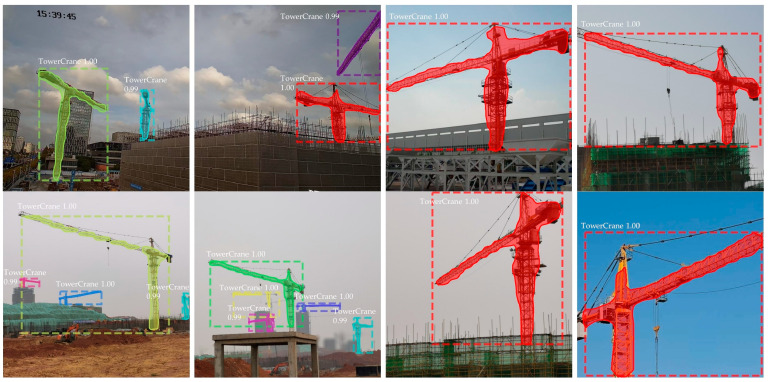
Examples of detection and segmentation images.

Figure 15 shows some of the detection and segmentation results, demonstrating that the instance segmentation performance fulfilled the requirements.

### 4.3. Performance of Feature Reconstruction and 3D Localization

The goal of geometric feature reconstruction is to extract the edge line segment of the tower crane and provide the features ${\left(\begin{array}{cc} l & \tau \end{array}\right)}^{T}$, where l is measured in pixels and τ is represented by a tangent value instead of a radian value. To evaluate the performance of the feature reconstruction, 100 manually labeled images were used and the results are shown in Table 4. According to the results, the average percentage error (APE) of l was 1.23% and the APE of τ was 1.69%, indicating that the performance of the geometric feature reconstruction fulfilled the requirements.

**Figure 16:**
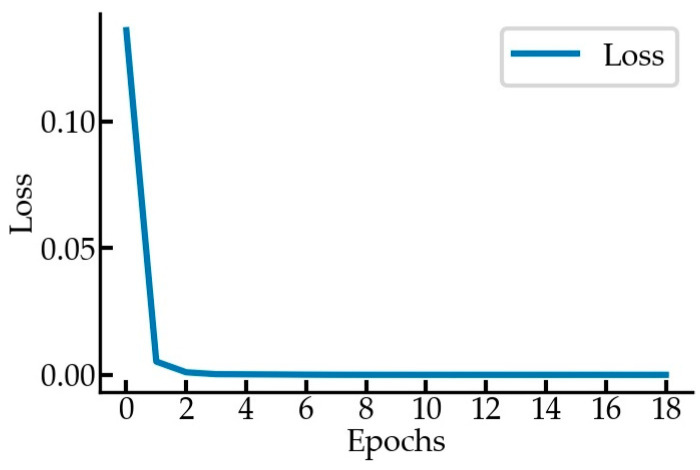
The loss curves for BPNN training.

In this paper, a BPNN was used to estimate the nonlinear relationship of ${\left(\begin{array}{cc} l & \tau \end{array}\right)}^{T}$-θ, with one input layer ${\left(\begin{array}{cc} l & \tau \end{array}\right)}^{T}$, one output layer θ, and two hidden layers containing 10 and 5 neurons, respectively. Figure 16 shows the loss curves. The coefficient of determination of the BPNN prediction was above 0.99, indicating good prediction performance.

**Figure 17:**
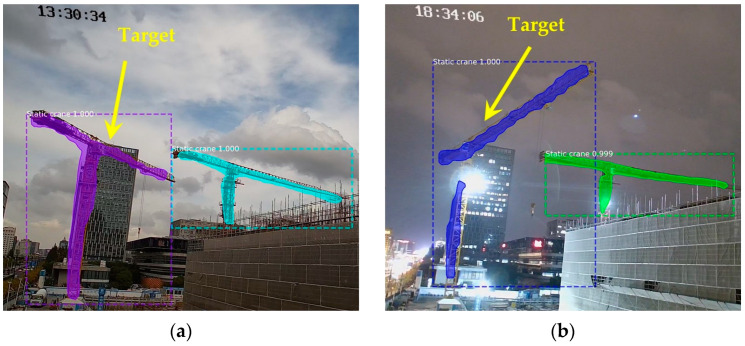
Different scenarios: (**a**) daytime; (**b**) nighttime.

Based on the obtained videos with fixed views, as shown in Figure 17 and Table 5, three video segments representing different scenarios were selected for comprehensive performance evaluation. All three video segments included complete jib rotation, trolley translation, and hook lifting and lowering movements. The ground-truth values of θ, r, and k were collected using inclinometers and distance sensors installed on the tower crane with a frequency of 1 s. From the information presented in Section 4.2, it is known that the frame rate of Mask-RCNN+ResNet101 can reach 6.23 FPS. As Mask-RCNN is the most time-consuming calculation module in this framework, it imposes an upper limit on the detection speed of the entire framework. Therefore, the FPS for evaluation in the selected video segments were set at 6, 3, and 2, respectively.

**Figure 18:**
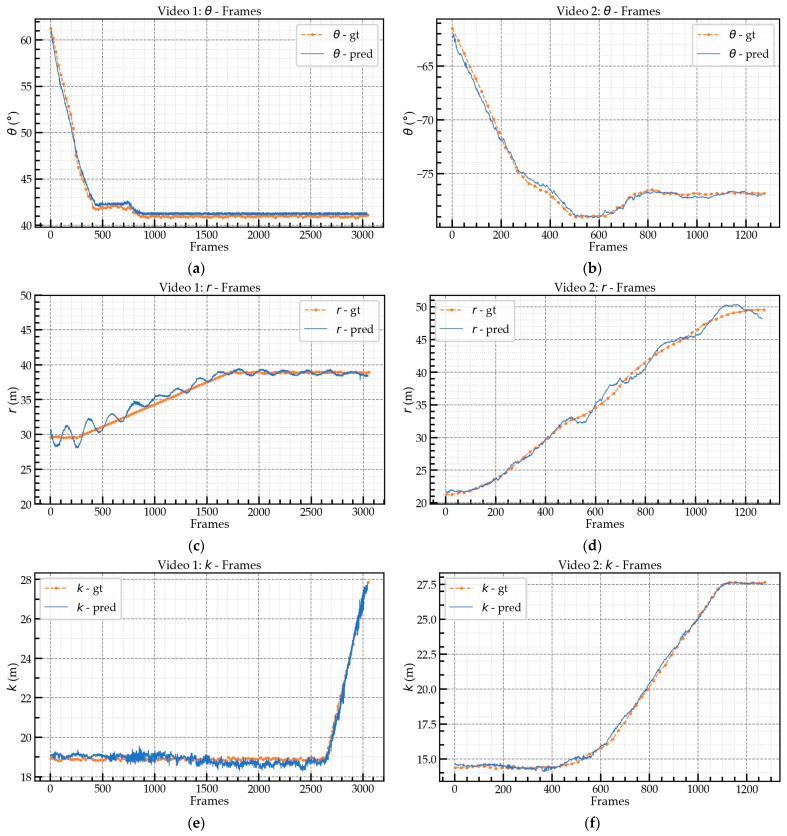
Prediction and ground-truth values for videos 1 and 2: (**a**) video 1, rotation angle θ; (**b**) video 2, rotation angle θ; (**c**) video 1, r; (**d**) video 2, r; (**e**) video 1, k; (**f**) video 2, k.

**Figure 19:**
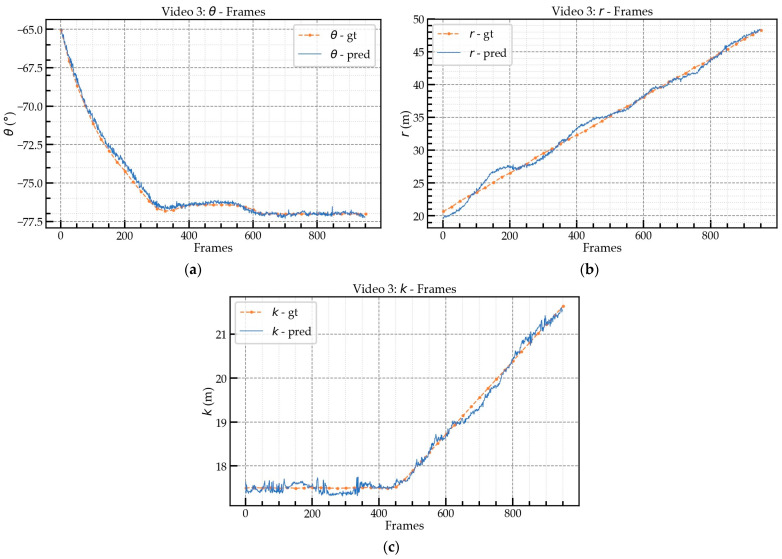
Prediction and ground-truth values for video 3: (**a**) video 3, rotation angle θ; (**b**) video 3, r; (**c**) video 3, k.

Figure 18 and Figure 19 illustrate the prediction (pred) and ground-truth (gt) values. Figure 18a,c,e represent the prediction values for the video 1 segment; Figure 18b,d,f represent the prediction values for the video 2 segment; and Figure 19 represents the prediction values for the video 3 segment. Table 6 shows the mean and maximum absolute errors (AEs) between the ground-truth and predicted values at the different FPS. The mean AEs of the θ predictions for videos 1, 2, and 3 were less than 0.9°, with videos 2 and 3 having AEs of approximately 0.8°. The average maximum AE for θ was about 1.5°. For r, the mean AE of video 1 was about 0.5 m, which was better than the mean AEs of videos 2 and 3. The performance of the r prediction for videos 2 and 3 was similar, with a mean AE of about 0.6 m. The k prediction achieved high accuracy for all three videos and was better than the r prediction due to the slight swing of the hook during movement, which led to the deviation in the r prediction. Regarding the θ prediction, when the angle between the jib and the camera’s optical axis $Z_{C}$ was smaller, the AE was larger because when the angle between the jib and the camera’s optical axis $Z_{C}$ became smaller, the same degree of movement caused a greater change in the pixels, i.e., the sensitivity of the extrinsic matrix. In particular, video 3 was tested at nighttime (18:00) and also achieved high accuracy. However, the performance for the k prediction for video 3 was not as good as that for videos 1 and 2 due to the small size of the hook, which led to unstable bounding box detection by Mask-RCNN in low illumination conditions. The FPS had little effect on the mean AE of the predictions but it did have an impact on the maximum AE.

**Figure 20:**
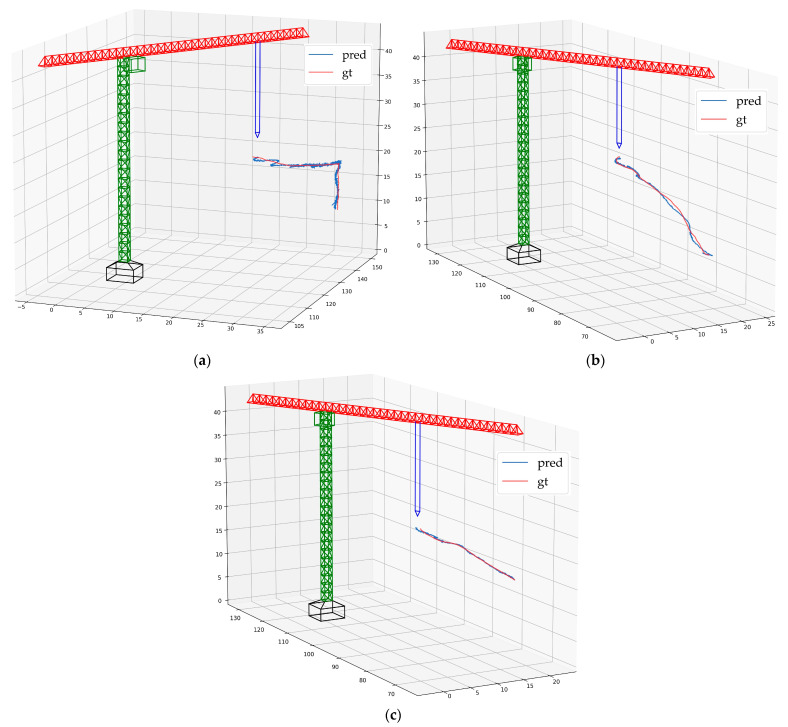
The 3D trajectory of the hook: (**a**) video 1; (**b**) video 2; (**c**) video 3.

Figure 20 illustrates the 3D trajectory of the hook’s motion. The predicted trajectory shows a high degree of proximity with the ground-truth trajectory. In Figure 20a, there is a deviation between the predicted trajectory and the ground-truth trajectory, which is due to the slight swing of the hook during the acceleration phase.

## 5. Discussion and Conclusions

This study proposes a framework that combines multiple advanced vision-based methods (Mask-RCNN, MLSD, and gc-horizon-detector) to perform the real-time 3D localization of tower cranes using a monocular far-field camera, including the jib orientation and the hook position. The real-time 3D localization of tower cranes can aid safety monitoring on construction sites and for crane operators, thereby reducing the occurrence of collision accidents. Compared with previous vision-based tower crane monitoring methods, this study utilizes existing far-field surveillance cameras on construction sites, making them more versatile. Previous studies rarely estimated the jib rotation angle using vision, whereas this study proposes a method to estimate the jib rotation angle using vision, achieving good accuracy. Therefore, the main contribution of this work is to explore the integration of state-of-the-art CVB methods to achieve and promote the digitalization and automation of construction site management and provide a more universal framework.

This paper makes three main contributions. First, it utilizes existing far-field surveillance cameras, providing a non-invasive method to enhance safety on construction sites. Previously, methods for the 3D localization of the hook used cameras installed on the jib to shoot downwards vertically, which had various limitations. The far-field camera used in this study has a larger field of view. By using prior knowledge of construction site landmarks and horizon detection, combined with projective geometry techniques, the extrinsic parameters of the camera are obtained. The results show that the prediction error of the extrinsic parameters is less than 5.0%, and the error of the most important parameter, the camera height, is only 1.9%. These results provide a reliable basis for monocular 3D localization.

The second contribution is the proposal of a more refined method for object pose estimation. Mask-RCNN, which is an advanced visual method, can detect objects and perform pixel-level instance segmentation. Previous research mainly focused on objects with regular shapes such as rectangles and circles, whereas tower cranes have a unique shape. This study proposes a method based on pixel masks and line analysis to extract the geometric pose features of tower cranes. The detection results of Mask-RCNN are close to those of the benchmark. The error of geometric feature reconstruction is less than 2.0%, demonstrating the good performance of the network.

The third contribution is the establishment of a relationship between the geometric features and 3D coordinates using a BPNN. The accuracy and error of the method were verified using three video segments. The experimental results show that the prediction method has high accuracy at the different FPS. The mean AE of the jib rotation angle estimation is less than 0.9° for all three videos, whereas the mean AEs of the *r* and *k* predictions are less than 0.7 m and 0.4 m, respectively. These results fulfill the requirements for the use of tower cranes on construction sites.

There are also some limitations of this study. Firstly, when the jib is partially obscured, this study can still accurately perform detection. However, when the jib is mostly obscured, it becomes difficult to extract geometric features based on pixel masks and line analysis. For example, when the building height obstructs the view of a far-field camera, it is difficult to extract effective geometric features. One possible solution is to perform 3D localization from different fields of view using all the surveillance cameras on the construction site, which, together, form a surveillance network. It is also possible to manually adjust the positions of the cameras in different stages of construction.

Secondly, during the acceleration phase, due to the flexibility of the wire rope, the hook will swing slightly, which does not conform to the calculation assumptions, resulting in distorted 3D localization. Future efforts will focus on finding a better method for the 3D localization of the hook such as GPS or laser scanning.

Based on the research presented in this paper, there are two future research directions. The first is to construct a real-time warning system for the crane commander/driver to monitor collision risks on the construction site. The second is to analyze production progress by locating the hook. The layout of the site represents its working status. Locating the hook will help to analyze production progress and improve production efficiency.

## Figures and Tables

**Table 1:** Localization of objects in different scenarios.

Scenarios	Limitations
Ground-plane objects	Difficulties in obtaining extrinsic parameters. Simplified calculation based on plane assumption and affine transformation. Multiple cameras needed for 3D pose estimation.
Aerial objects	Special view angle of imaging. Lack of generality in calculation methods. Combination with markers. Issues with installation of additional equipment.

**Table 2:** Accuracy of camera’s extrinsic parameters.

Extrinsic Parameters	Ground Truth	Estimation	Percentage Error ^1^ (%)
Camera height, h(m)	11.08	11.29	1.90
Pitch angle, α (rad)	−0.1934	−0.1854	4.14
Yaw angle, β (rad)	0.2293	0.2380	3.79
Roll angle, γ (rad)	0.0405	0.0399	1.48

^1^ Percentage Error = |gt − est|/gt × 100%.

**Table 3:** Results of trained Mask-RCNN and benchmark.

Metrics	Tower Crane (%)	Hook (%)	Benchmark Results (%)
AP^IoU=0.50^	72.42	85.41	72.74 ^1^
AP^IoU=0.75^	46.93	47.13	47.99 ^1^
mAP	40.64	47.20	45.35 ^1^

^1^ Value from Ref. [69].

**Table 4:** The performance of the geometric feature reconstruction.

Line	l (Pixel)		τ	
	est	gt	est	gt
1	415.15	418.27	0.5960	0.6012
2	566.56	563.72	0.4057	0.3989
3	620.35	624.76	0.3590	0.3699
…	…	…	…	…
99	635.24	630.75	0.3487	0.3406
100	470.74	465.06	−0.4579	−0.4643
Percentage Error	1.23%		1.69%	

**Table 5:** Statistics of the video segments for implementation.

Video	FPS	Resolution	Length	Frames	Illumination
1	24	2560 × 1440	127 s	3045	daytime
2	24	2560 × 1440	53 s	1267	daytime
3	24	2560 × 1440	39 s	949	nighttime

**Table 6:** Absolute errors (AE) for videos 1, 2, and 3.

Video	FPS	θ (°)		r (m)		k (m)	
		Mean AE	Max AE	Mean AE	Max AE	Mean AE	Max AE
video 1	6	0.8692	1.6340	0.5371	1.9608	0.2073	0.8478
	3	0.8763	1.5072	0.5364	1.7860	0.2123	0.8478
	2	0.8711	1.5381	0.5410	1.7721	0.2125	0.6662
video 2	6	0.8058	1.3852	0.6329	1.7776	0.1437	0.5309
	3	0.8109	1.3852	0.6399	1.7151	0.1452	0.4804
	2	0.8008	1.3852	0.6296	1.7296	0.1447	0.4803
video 3	6	0.8641	1.6072	0.6324	1.7235	0.3865	0.7452
	3	0.8704	1.5853	0.6283	1.7236	0.3873	0.7451
	2	0.8580	1.5853	0.6298	1.7236	0.3859	0.7452

## Data Availability

Not applicable.
